# Zoonotic Helminth Diseases in Dogs and Dingoes Utilising Shared Resources in an Australian Aboriginal Community

**DOI:** 10.3390/tropicalmed3040110

**Published:** 2018-10-08

**Authors:** Felicity A. Smout, Lee F. Skerratt, Christopher N. Johnson, James R. A. Butler, Bradley C. Congdon

**Affiliations:** 1 One Health Research Group, College of Public Health, Medical and Veterinary Sciences, James Cook University, Townsville, QLD 4811, Australia; lee.skerratt@jcu.edu.au; 2 College of Science and Engineering and Centre for Environmental and Sustainability Science, James Cook University, Cairns, QLD 4870, Australia; brad.congdon@jcu.edu.au; 3 School of Biological Sciences, University of Tasmania, Private Bag 55, Hobart, TAS 7001, Australia; C.N.Johnson@utas.edu.au; 4 CSIRO Land and Water, EcoSciences Precinct, GPO Box 2583, Brisbane, QLD 4001, Australia; James.Butler@csiro.au

**Keywords:** dingo, dogs, aboriginal, diseases, canine, zoonoses

## Abstract

The impacts of free-roaming canids (domestic and wild) on public health have long been a concern in Australian Indigenous communities. We investigated the prevalence of zoonotic helminth diseases in dogs and sympatric dingoes, and used radio telemetry to measure their spatial overlap, in an Aboriginal community in the Wet Tropics of Australia. Samples collected from dingoes and dogs showed high levels of infection with the zoonotic hookworm, *Ancylostoma caninum*. Dingoes were also positive for *A. ceylanicum* infection (11.4%), but dogs were infection free. Whipworm, *Trichuris vulpis,* infection was far more prevalent in necropsies of domestic dogs (78.6%) than dingoes (3.7%). Dogs were free from *Dirofilaria immitis* infection, while dingoes recorded 46.2% infection. Eleven dingoes and seven free-roaming domestic dogs were fitted with Global Positioning System collars and tracked over an extended period. Dingo home-ranges almost completely overlapped those of the domestic dogs. However, dingoes and dogs did not utilise the same area at the same time, and dogs may have avoided dingoes. This spatial overlap in resource use presents an opportunity for the indirect spill-over and spill-back of parasites between dogs and dingoes. Tracking and camera traps showed that the community rubbish tip and animal carcasses were areas of concentrated activity for dogs and dingoes.

## 1. Introduction

Globally, the risk of disease transmission between free-roaming dogs (*Canis familiaris*), wildlife, and humans is a growing concern, driven largely by the burgeoning population of domestic dogs [1,2,3]. In Australia, free-roaming canids and the potential public health issues associated with them have long been a concern in Indigenous communities. These animals can include unrestrained domestic dogs from within the community, along with wild dogs and dingoes (*Canis dingo* Meyer, 1793) from surrounding areas. Urban expansion into previously undeveloped areas around communities has brought people and their pets into closer contact with dingoes, resulting in antagonistic interactions and stakeholder conflict [4]. One potential interaction is the transmission or ‘spill-over’ of diseases, including zoonotic parasites, from dingoes to dogs and thus to humans. Investigating all potential hosts and their interactions is hence necessary to understand and mitigate ‘spill-over’ and ‘spill-back’ of zoonotic infection [5]. 

It is estimated that soil-transmitted helminths infect two billion people worldwide, with many of these infections occurring in Australia’s closest neighbours, South-East Asia [6]. Some of the parasites of zoonotic importance in the Wet Tropics bioregion of northern Queensland include the common roundworm, *Toxocara canis*, and the dog hookworm, *Ancylostoma caninum*. These worms can cause ‘visceral larval migrans’ and ‘cutaneous larval migrans’, respectively, in humans, along with a range of symptoms. Shield et al. [7] found a prevalence as high as 28% and 21% for *Strongyloides stercoralis* and *T. canis* infection, respectively, in children and adults in a remote Aboriginal community of the Northern Territory. The recent finding that *Ancylostoma ceylanicum* occurs in domestic dogs [8,9], dingoes [10], and humans [11] in Australia is of further concern, due to the much higher prevalence of this parasite than previously thought [6] and its potential to cause patent infections, resulting in significant illness in humans [12,13].

Another recent study in Queensland has revealed that dingoes are a reservoir host for heartworm (*Dirofilaria immitis*) in low-density housing areas [14]. Heartworm is spread by mosquito vectors and is a potentially life-threatening disease for canines. Although infection in humans is generally asymptomatic, the parasite can cause infarction and nodule formation in the lungs [15,16].

Understanding the interactions between the multiple hosts and vectors of canid diseases in and around human settlements is critical to public health management [17,18]. Given that many helminth diseases are often spread through the faecal-oral route, faecally-contaminated areas of mutual resource use may be important locations for disease transmission via exposure to shedding pelage and faeces, even when no direct contact has taken place [19,20].

Traditionally, dingoes accompanied Aboriginal people as hunting aides, companions, and protectors. Dogs have replaced dingoes in most communities and, to some extent, hold the same sacred position [21]. Due to this complex relationship, dogs are often not restrained to house yards and are given the freedom to come and go as they please. Veterinary services for Indigenous communities are limited [18], have offered little chemoprophylactic therapy for community dogs, or education about dog population management or disease risks, and have involved inappropriate mass culling of dogs without informed consent from animal owners [22]. The result of this has often been a quick return to pre-cull numbers and a loss of trust in agencies responsible for the management of dogs.

The aim of the present study was to determine the prevalence and intensity of helminth parasite infection of domestic dogs and dingoes in a Wet Tropics Indigenous community and identify ‘hot-spots’ for potential transfer of disease by evaluating the spatial and temporal use of habitat and resources by sympatric dogs and dingoes. We use our results to identify interventions that may reduce the risk of disease transmission between hosts, and thus to improve public health management. These results can then be extrapolated to other Indigenous communities in northern Australia to better manage free-roaming dog and dingo populations, and broader biosecurity risks and threats.

## 2. Materials and Methods

### 2.1. Study Area

**Figure 1:**
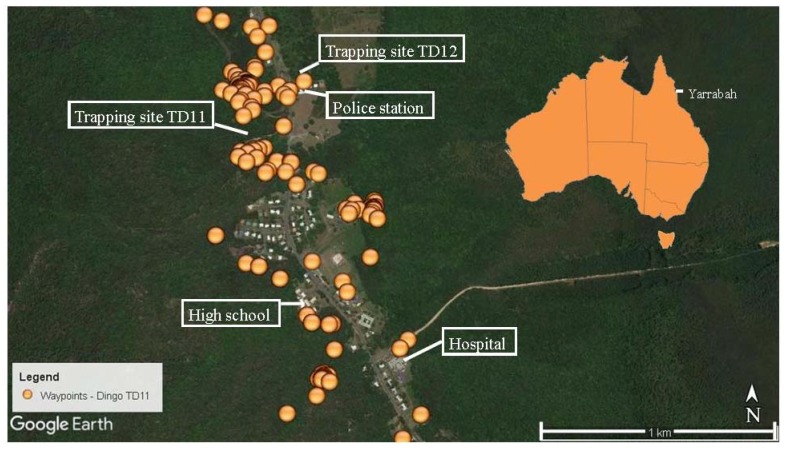
Waypoints collected in Yarrabah over a seven-day period (7 November 2013 to 14 November 2013) for female dingo TD11.

The study was conducted in and around the Yarrabah Aboriginal community, in the rural lowlands of the Wet Tropics bioregion in north-eastern Queensland, Australia (Figure 1). This bioregion contains remnant rainforest, which holds globally-significant biodiversity and cultural values. These values are recognised by its designation as the Wet Tropics World Heritage Area (WTWHA) [23,24]. Yarrabah is 53 km by road from the regional city of Cairns. It has a tropical climate, with strongly seasonal rainfall of approximately 2000 mm per annum. Mean monthly temperatures range from 20 °C to 29 °C. The community is bordered by ocean on two sides and Mount Yarrabah, with an elevation of 602 m to the north-west. Vegetation is varied, ranging from estuarine mangroves to coastal swamps of melaleuca, eucalyptus, and palm species. The study area covered approximately 160 km^2^ and is bordered by, and includes, the State Forest and National Park. 

Yarrabah has a population of 2346 people [25]. This community was chosen due to its proximity to a known dingo population, as well as being representative of other Indigenous communities in the Wet Tropics and elsewhere in northern Australia. 

### 2.2. Dingoes

#### 2.2.1. Dingo Necropsy Samples

Twenty-seven dingo carcasses (15 males and 12 females) were supplied by Cairns Regional Council animal control officers and local landholders following routine control measures from 2007 to 2012. These dingoes originated from farmland adjacent to Yarrabah as well as from the outer suburbs of northern and southern Cairns and Atherton. No animals were killed specifically for this study. All specimens were bagged and frozen as soon as possible following collection, and information regarding the collection date and location were noted.

The stomach and intestines were excised and all contents were washed thoroughly and preserved in 70% ethanol for later microscopical examination. The heart and lungs were also excised and the right ventricle and pulmonary arteries were examined for the presence of *D. immitis.* Further details, such as age, sex, and body condition score (Purina Body Condition System™ 1-9, Nestlé Purina Pet Care Center, Rhodes, NSW, Australia), were recorded.

#### 2.2.2. Dingo Trapping

Twelve dingoes (seven females and five males) were trapped either in the outer suburbs of the Cairns/Gordonvale region adjacent to Yarrabah, or within the Yarrabah community and fitted with Global Positioning System (GPS) tracking collars. 

Ten of the twelve dingoes were trapped using Oneida Victor® Soft Catch® traps (Oneida Victor Inc. Ltd., Euclid, OH, USA) during a concurrent study undertaken in the area [26]. Traps were monitored using Trapsite VHF transmitters (Telonics, Mesa, AZ, USA) attached to the trap chain. Trap sites were also visually inspected each morning.

Two further dingoes, one female and one male, were trapped within the Yarrabah community on November 2012 and May 2013, respectively. Due to the presence of many wandering domestic dogs, large cage traps were used here. Traps were placed in shaded areas, baited with raw chicken carcasses, and visually inspected at least twice daily. 

#### 2.2.3. Processing Dingoes

An intra-muscular injection of Domitor® (medetomidine hydrochloride, Pfizer Australia Pty. Ltd., West Ryde, NSW, Australia) was used to sedate the animals. To further reduce risk to field workers, dingoes were placed on a restraint board with straps over their neck and loins and fitted with a muzzle. Animals were examined for the presence of external parasites and faecal samples were collected. Age and body condition scores were estimated and animals were weighed (Shimano 45 kg stainless weighing scale). Five mL of blood was collected from the jugular vein of each animal and stored in ethylene diamine tetraacetic acid (EDTA) tubes and refrigerated at 4 °C for antigen testing within 24 h. Animals were implanted with a microchip (Trovan 956 ISO, Microchips Australia Pty. Ltd., Keysborough, Australia) on the dorsal midline between the shoulder blades using a 12-gauge implanter needle (Trovan Deluxe [IME] Implanter, Microchips Australia Pty. Ltd., Keysborough, Australia). 

#### 2.2.4. GPS Tracking

All dingoes were fitted with a Tellus™2A GPS tracking collar (Followit AB, Lindesberg, Sweden). Following processing, the sedative was reversed with Antisedan^®^ (atipamezole hydrochloride, Pfizer Australia Pty. Ltd., West Ryde, NSW, Australia) and the dingoes were monitored until they moved away from the trap site.

GPS collars were programmed to record a location (waypoint) every two hours for 14 days (alternating between odd and even hours at one-week durations), and then every 10 min for half a day (alternating between before noon and after noon each time) and every two hours for the rest of the day on the 15th day [26].

The collars also included a release mechanism that could be manually activated via a remote communication device (RCD-04, Followit AB, Lindesberg, Sweden); this device was also used to remotely download data from collars. A timed-release mechanism was set to release 10 months after deployment for collars not manually released. GPS data were screened to remove location errors by removing two-dimensional (2-D) locations with a positional dilution of precision (PDOP) <5 [26,27].

Maps were created by entering waypoints into Google Earth using Excel to Keyhole Markup Language (KML) file through Earth Point Tools for Google Earth 2017/2018 (http://www.earthpoint.us/ExcelToKml.aspx).

### 2.3. Domestic Dogs

#### 2.3.1. Faecal and Tissue Samples

Fifty faecal samples were collected from free-ranging domestic dogs within the Yarrabah urban area. Heart, stomach, and intestinal samples were collected from 28 domestic dog carcasses (9 males and 19 females), supplied by Yarrabah Aboriginal Council Animal Control Officers between December 2010 and December 2011. All specimens were necropsied, as per Section 2.2.1 above, immediately following euthanasia.

#### 2.3.2. Domestic Dog Tracking

Seven domestic dogs (two females and five males) were recruited from three households within the community and fitted with motion detector GPS-data loggers (i-gotU GT-600, Mobile Action Technology, Taipei, Taiwan) attached to a 40 mm wide synthetic collar. The dimensions of the loggers were 46 × 41.5 × 14 mm with a weight of 37 grams. Individual loggers were further protected and waterproofed by being wrapped inside plastic film, placed inside a plastic zip lock bag, and then covered with black heatshrink tubing. 

Data loggers were set to record a position every 41 seconds and were changed twice per week to retrieve maximum data per animal. The battery life on trackers was between two and five days with four days being the usual case. Community dogs were tracked from October 2012 to September 2013. Movements were observed and mapped on Google Earth. The trapping site for TD11 and the rubbish tip were monitored with camera traps over a six-month period (PC900 HyperFire™, Reconyx, Holmen, WI, USA and DLC Covert II, Covert Scouting Cameras Inc., Lewisburg, KY, USA).

### 2.4. Parasitology Techniques

#### 2.4.1. Detection of Adult *D. immitis* antigen

Blood samples from trapped animals were tested for circulating antigen, as per the manufacturer’s instructions, using a commercial ELISA SNAP test kit for heartworm (IDEXX Laboratories Inc., Rydalmere, NSW, Australia): Sensitivity, 84%, and specificity, 97% [28]. The IDEXX SNAP test detects the glycoprotein found in the reproductive tract of the mature female *Dirofilaria immitis* worm, thus further tests, as described below, were undertaken to ensure those animals with low, or male only, worm burdens were not missed.

#### 2.4.2. Blood Smears

Thin blood smears from the 12 dingoes were also stained with DiffQuik and examined under a light microscope to further test for the presence or absence of microfilariae. Where samples returned an antigen-negative result and no microfilaria were seen on a stained slide, a further test was performed by microscopically investigating for microfilariae in the region above the buffy coat of a spun blood sample in a microhematocrit tube [29]. *Dirofilaria immitis* microfilariae were identified morphologically and distinguished from *Acanthocheilonema* (syn. *Dipetalonema*) *reconditum*, a filarial parasite of the subcutaneous tissues and fascia of canids, according to existing descriptions [30,31]. *Dirofilaria repens* is not known to occur in Australia [32].

#### 2.4.3. Faecal Examination

Faecal samples (5 g if available) were examined macroscopically for the detection of proglottids and then screened by direct microscopy for the presence of worm eggs and larvae. Further molecular analysis in the form of direct PCR was undertaken on all 50 of the dog faecal samples and eight of the 12 dingo samples (66.7%) to determine *Ancylostoma* species present. DNA sequencing was conducted on all positive PCR samples [10,33].

#### 2.4.4. Microscopic Examination

Intestinal contents were examined under dissecting and compound microscopes. Positive identification of *Ancylostoma* spp. was established using those criteria documented by Biocca [34]. Where heavy infections were present, at least fifty individual hookworms were identified before recoding the *Ancylostoma* spp. present. As dogs were housed together prior to euthanasia, ectoparasite prevalence was not focused on in this study; however, microscopic examination was used to identify fleas, ticks, and mites seen.

### 2.5. Data Analysis

Prevalence of infection was calculated on all dingo and dog samples by dividing the number of samples positive for parasite infection by the total number of samples collected. Home range was estimated using 100% minimum convex polygon (MCP100) [35]. The area of the polygon was measured using Google Earth co-ordinates and entering data into Earth Point Tools for Google Earth 2017 (http://www.earthpoint.us/Shapes.aspx).

### 2.6. Ethics

All protocols were reviewed and approved by James Cook University Human and Animal Ethics Committees (Approval nos. H4264, A1495 and A1546).

## 3. Results

### 3.1. Parasite Infections

Twelve dingoes were trapped between October 2010 and May 2013. One dingo (TD05) was lost and not able to be tracked following release, but has been included in the parasite infection data (Table 1). All dingoes appeared to be well muscled and in a healthy condition although four lactating females were slightly underweight. Body condition scores ranged from three (underweight) to five (ideal). Faecal samples revealed 100% infection with *A. caninum*, with one animal (12.5%) returning a positive PCR result for *A. ceylanicum*. A similar result was also obtained for necropsied dingoes of 100% and 11.1% infection for *A. caninum* and *A. ceylanicum*, respectively (Table 1). Further molecular identification to differentiate *A. caninum* from *A. ceylanicum* was not undertaken on the faecal samples of four of the dingoes, as they were trapped after the completion of laboratory work. No skin lesions were seen on any dingoes.

The physical condition of domestic dogs varied. All collared dogs (*n* = 7) were well muscled and healthy (BCS = 5, ideal), however, the stray, necropsied animals (*n* = 28) ranged in body condition score from the minimum of one (very poor) to seven (overweight), with the majority scoring two–three (79%). Domestic dogs had an overall high prevalence of *A. caninum*, with 100%, 96.4%, and 88.0% infection in collared dogs, necropsied dogs, and domestic dog scats (*n* = 50), respectively (Table 1). *Ancylostoma ceylanicum* was not identified in domestic dogs. 

Similar levels of infection with *Toxocara canis* were seen in dingoes (41%) and domestic dogs (29.4%) overall, however, *Trichuris vulpis* and *Dipylidium caninum* infections were far more prevalent in domestic dog necropsies (78.6% and 71.4%, respectively) than in dingo necropsies (3.7% for both). The reverse was seen for *Spirometra erinacei* infections, with 46.1% and 3.5% overall prevalence in dingoes and dogs, respectively. It is also noted that dogs were free from *Dirofilaria immitis* infection, while dingoes had an overall infection rate of 46.2%. All trapped dingoes that returned positive antigen tests were also microfilariaemic positive (66.7%). No dingoes were antigen negative, but microfilariaemic positive. Many of the necropsied dogs (71%) also showed signs of chronic skin diseases from which *Sarcoptes scabiei* and *Demodex canis* mites were identified. *Ctenocephalides felis* and *C. canis* along with the common dog tick, *Rhipicephalus sanguineus*, were also found on dogs. 

### 3.2. Home Ranges and Resource Use

A total of 1253 and 308 tracking days were analysed for dingoes and domestic dogs, respectively. The mean home range size for dingoes was 44.8 ± 11.38 km^2^, and 2.3 ± 0.59 km^2^ for dogs (Table 2). Female dogs ranged further than male dogs, with a mean home range of 3.8 ± 1.35 km^2^ and 1.7 ± 0.50 km^2^, respectively. In contrast male dingoes had larger ranges than females, with home range averages of 56.2 ± 22.37 km^2^ and 38.3 ± 13.30 km^2^, respectively, but the difference was not significant (*t*-test: *t* = 0.7387, d.f = 9, *p* > 0.05). 

Of the 11 dingoes tracked, the two trapped within the Yarrabah community (TD11 and TD12) spent most their time there. One other female (TD09) trapped at Glen Boughton, approximately six km away, was recorded on the southern side of the community on one day only. TD11 and TD12 regularly entered the community and utilised areas around the police station, hospital, and high school (Figure 1).

**Figure 2:**
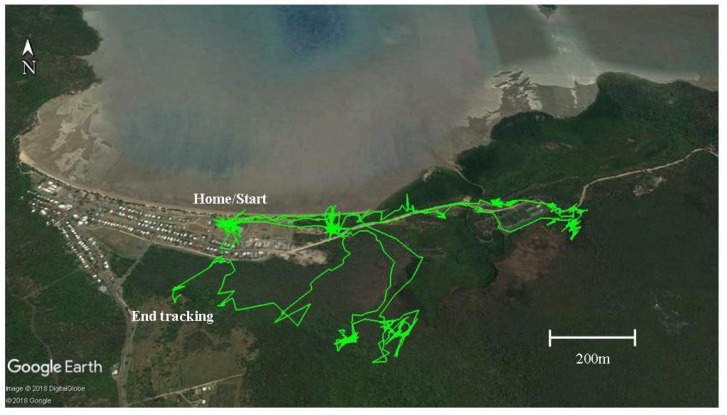
Track map from i-gotU GT-600 (Mobile Action Technology, Taipei, Taiwan) data logger for female domestic dog CD01 over 96 h in Yarrabah showing forays away from urban boundaries as viewed on Google Earth engine.

Five collared dogs from one household ventured out on forays almost daily, with many these trips to the rubbish tip for short periods. These five dogs spent a considerable amount of time, on average two to three hours per day, outside of urban boundaries. The i-gotU GT-600 (Mobile Action Technology, Taipei, Taiwan) data loggers enabled precise tracking of the dogs’ forays, which were viewed on Google Earth engine (Figure 2). 

Following the download of data showing a particularly long foray where all five dogs from the same household had visited a site for a whole day and then revisited for several days afterward, the first author investigated the site and found the remains of a pig carcass. It was impossible to determine how the pig had died due to decomposition and scavenging. Thus, it may have been shot and left by a local hunter or killed by dingoes. It was most unlikely that the dogs killed the pig as no high-speed chase was recorded prior to the dogs’ extended time at the site. The young male dingo (TD12) was also tracked in this area at a later date.

**Figure 3:**
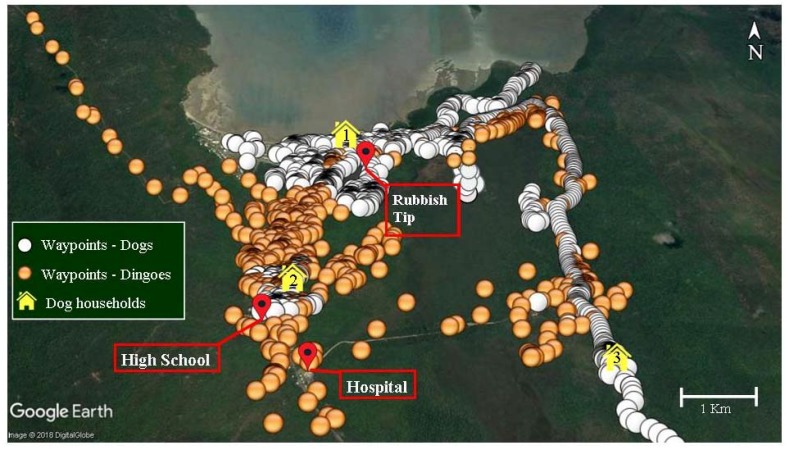
Waypoints for all tracked dingoes and dogs, plus key locations in and around Yarrabah.

A map of the community showing waypoints recorded for both dogs and dingoes, along with the home site for dogs, is shown in Figure 3. Many waypoints around the dogs’ homes have been removed to reduce crowding of those locations and to enable clearer viewing of dingo waypoints. Importantly, dingo home ranges almost completely overlapped that of the domestic dogs and dingoes spent a substantial amount of their time (98%) in areas used by dogs.

The camera traps situated at the trapping site for TD11 (Figure 1) and the rubbish tip (Figure 3) captured images of dingoes and dogs, plus horses (*Equus caballus*) and people. Although there was spatial overlap in resource use, the recorded waypoints along with captures by the trap cameras showed no overlap in the times study dogs and dingoes utilised the same areas. There was no discernible temporal pattern seen by dogs or dingoes utilising a specific habitat. Dogs, dingoes, horses, and people were all seen both day and night and domestic dogs were often seen travelling with people.

## 4. Discussion

Our assessment of potentially zoonotic helminth parasites indicated widespread infections in dingoes and dogs from an Indigenous community in the Wet Tropics. The dog hookworm, *A. caninum*, was the most prevalent parasite in both Yarrabah dogs and dingoes in the region, infecting 91.8% and 100%, respectively. The high prevalence of *A. caninum* was similar to the most recent findings by Slapeta et al. [36] on infections in free-roaming dogs in Yarrabah (100%). Reports from other parts of Australia indicate a moderate to low prevalence [37,38]. Studies indicate that enteric infection with *A. caninum* is a leading cause of human eosinophilic enteritis in north eastern Australia [39,40].

Although *A. ceylanicum* was not identified in the dogs in this community, a previous study did find soil samples positive for *A. ceylanicum* in the area [9]. Unfortunately, the faecal samples of the two dingoes trapped within the community were not investigated with further molecular tools; however, these animals were positive for strongyle-type eggs and *A. ceylanicum* was found in four other dingoes in this study and has been found previously in dingoes and community dogs in Far North Queensland [9,10]. This parasite is of particular concern as it can cause a patent infection in humans [11].

A further five helminths were found comprising three nematodes and two cestodes. *Trichuris vulpis* was detected in 48.2% of dogs and 5.1% of dingoes. This canine whipworm has been reported as common in domestic dogs in urban environments of Australia [41,42], but appears to be uncommon in dingoes. Intermittent shedding of this parasite in faeces, along with the methodology used for detection, may be the reason a lower prevalence was detected in faecal examination (31.3%) and versus necropsy (78.6%) in dogs. An ongoing debate exists regarding the zoonotic potential of *T. vulpis* and its potential to cause visceral larva migrans in humans, although cases of presumed *T. vulpis* infection in humans have been described [43] along with studies using molecular techniques to speciate infection in humans [44,45].

Previous reports show a lower prevalence of *T. canis* in other dog populations, both domestic and wild, compared with the present study of 29.4% and 41% overall in dogs and dingoes, respectively. Wallner [37] reported a prevalence of 12% in Townsville pound dogs and Coman [46] recorded a 13.5% prevalence in wild dogs in Victoria. Human infection of *T. canis* can result in fever, malaise, abdominal pain, wheezing, asthma and, in extreme cases of the ocular form, total blindness [47,48]. 

Canine heartworm, *D. immitis*, was found in 10 necropsied dingoes (37%) and eight (66.7%) of the trapped dingoes. The two dingoes trapped in the Yarrabah community both returned negative antigen test results and all domestic dog necropsies were free of infection. The absence of this parasite in community dogs may be a consequence of the ongoing use of ivermectin for the treatment of mange and heartworm [49]. Speare [50] found an initial prevalence of 87% of heartworm in dogs over one year of age in Yarrabah. Following a 12-month ivermectin program, the prevalence of heartworm had fallen to just 14%. The recent findings of Smout et al. [14] of a prevalence of 72.7% in dingoes in the region suggests that this disease is a continuing threat. Therefore, it is important that the results of this study do not lead to complacency in the community. 

The tapeworm, *Spirometra erinacei*, detected in dingoes (46.1%) is the second report of *S. erinacei* in dingoes in North Queensland, and suggests that the tapeworm is now established in dingoes in the area and likely reflects their diet and a suitable habitat for intermediate hosts [51]. *Dipylidium caninum* was detected in 71.4% of dogs and 3.7% of dingoes on necropsy. No infection was seen in faecal samples collected from either dogs or dingoes. Difficulty in finding proglottids in faeces may be the reason, along with the time elapsed between when scats were deposited and collected. Proglottids are frequently motile and may have moved away from the sample before it was collected. Symptoms of dipylidiasis infection in infant humans have been reported, but are generally rare and are described as mild and nonspecific [52].

The absence of *Echinococcus granulosus* in our study could be due to several reasons, including the test methodologies used, which are considered poor at recovering cestode eggs from faecal samples, although it should be noted that this parasite was also absent from necropsied animals. A previous study has also reported the absence of this parasite in domestic dogs in the Yarrabah community [50]. Generally, dingoes have a higher mortality rate near urban areas due to culling by local vertebrate pest control officers, which leads to disruptions in social organisation (pack size, average age, and hunting experience), causing dingoes to rely on smaller prey, such as rats and bandicoots (*Permales nasuta*, *Isoodon macrourus*) [26,53]. Thus, their diet contains fewer macropods and transmission rates of *E. granulosus* are reduced [54]. 

The large tapeworm, *Taenia hydatigena*, which is reported as common in wild dogs in Australia [41], was also not found in any dingoes or dogs in our study. These results further suggest that ruminant livestock may not be an important component of the diet of dingoes in the Cairns area, as found by Morrant et al. (2017a; 2017b) who, in a concurrent study, analysed diet samples collected from the dingoes necropsied here along with others in the region and found only one out of 269 samples (0.004%) contained domestic bovine, and one contained goat. A pocket of high cystic echinococcosis infection in cattle has been found previously on the Atherton Tableland, approximately 50 km inland from Cairns and at an elevation of 800 m [55].

Although the present study did not investigate ectoparasites due to captured dogs being housed together at the council pound before euthanasia, many of the dogs necropsied showed signs of chronic skin diseases due to *S. scabiei* and/or *D. canis* mite infection. Fleming et al [56] reported sarcoptic mange as a widespread disease in dingo populations throughout Australia; however, no skin lesions were seen on any of the dingoes (*n* = 39) in this study. The absence of infection in our study could be an indication that sarcoptic mange may be rare in dingoes in the Wet Tropics. 

The risk of transmission of parasite infections is likely to vary both temporally and spatially depending on host behaviours and transmission pathways. Our results show that dingo home-ranges almost completely overlapped those of domestic dogs although at no time were dingoes and dogs recorded together, supporting previous findings that dogs avoid dingoes [4]. During the study period, a community member witnessed two large (25–30 kg) male domestic dogs killed by three dingoes. The dogs had been chasing two young foals when they were attacked by the dingoes. The encounter took less than five minutes, whereupon the dingoes returned into the surrounding bush. The first author viewed the dog carcasses, finding both animals had deep lacerations to their throats and numerous other puncture wounds. Similar reports of aggression and predation by dingoes on domestic dogs are also common in other areas of the Wet Tropics and elsewhere in Australia, particularly on the boundary of rural and sub-urban settlements [4,57].

As dingoes and dogs appeared to avoid direct contact, transmission of disease would generally occur indirectly through faecally-contaminated areas that are mutually used for resources, such as food. The tropical climate of the region promotes parasite survival in the environment, particularly for soil-transmitted helminth infections, and hence allows for elevated parasite transmission and burdens throughout the year [58]. Further investigations using specialised techniques and concentrating on parasites, such as *Strongyloides stercoralis*, should be undertaken in the region.

Previous studies tracking the home ranges of domestic dogs from Aboriginal communities have reported average home ranges of 9.7 km^2^ (927 ha) for wandering dogs [59]. Durr and Ward [60] stated that dogs that roamed furthest were of most relevance for infectious disease transmission because they could potentially vector disease over greater distances. Sparkes et al. [61] found that male dogs tended to range over significantly larger areas than females. The dogs in our study had an average home range of 2.3 ± 0.59 km^2^ (230 ha) thus falling between the wandering and sedentary categories used by Meek [59]. 

The two dingoes trapped within the community (TD11 and TD12) had smaller home ranges than those exhibited by the other trapped dingoes [26]. This may be because they largely utilized anthropogenic resources not available to those dingoes outside the community setting. The young male dingo (TD12) was tracked to the local rubbish tip on numerous occasions, suggesting that he may have been scavenging food from the area. Five of the collared dogs also frequented the rubbish tip on an almost daily basis. This overlap of home ranges, and indeed dietary niches, increases the risk of disease transmission.

While there is a background risk of indirect transmission of parasites over the entire area where dingo and dog home ranges overlap, the primary risk is likely to be at locations where dog and dingo activity is concentrated. Mitigating strategies should include exclusion fencing of the rubbish tip, effective disposal of animal carcasses, public education about local zoonotic diseases and their prevention, and regular chemoprophylactic therapy of community dogs. Further studies utilising spatial statistical models would be invaluable to refine home-range and behavioural characteristics.

## 5. Conclusions

In summary, there is an elevated risk of transmission of canine parasitic diseases of zoonotic importance given the elevated parasite burdens in dogs and dingoes, and also on the substantial overlap of spatial habitat use and food resources of dingoes and domestic dogs, even within busy community areas. Hot spots of transmission between dingoes, dogs, and humans are likely to be sources of anthropogenic-derived food, such as the rubbish tip, animal carcasses, and the high school sporting grounds. 

Further investigations on transmission risks for canine infectious diseases in other Indigenous communities are necessary to identify appropriate mitigation strategies. For animal disease control programs to have a sustainable outcome, it is vital that extensive consultations with local environmental health officers and animal management workers are undertaken throughout the process.

## Figures and Tables

**Table 1:** Number of infected and prevalence of helminth infections in free-roaming domestic dogs and dingoes.

(% Prevalence)	Necropsy Dingo ^†^	Collared Dingo *	Total Dingoes	Necropsy Dog ^†^	Collared Dog *	Dog Scats *	Total Dogs
	(*n* = 27)	(*n* = 12)	(*n* = 39)	(*n* = 28)	(*n* = 7)	(*n* = 50)	(*n* = 85)
**Nematoda**							
*Ancylostoma caninum*	27 (100) (91–100) ^§^	8/8 (100) 4NI (78–100)	35/35 (100) (93–100)	27 (96) (86–100)	7 (100) (77–100)	44 (88) (78–98)	78 (92) (85–98)
*Ancylostoma ceylanicum*	3 (11) (0–24)	1/8 (13) 4NI (0–38)	4/35 (11) (0–23)	0	0	0	0
*Toxocara canis*	12 (44) (27–62)	4 (33) (9–58)	16 (41) (26–56)	13 (46) (29–64)	2 (28.6) (0–58)	10 (20) (9–31)	25 (29) (20–39)
*Trichuris vulpis*	1 (4) (7–14)	1 (8) (0–28)	2 (5) (0–14)	22 (79) (63–94)	2 (28.6) (0–58)	17 (34) (21–47)	41 (48) (38–59)
*Dirofilaria immitis*	10 (37) (20–55)	8 (67) (43–91)	18 (46) (31–61)	0	NI	NI	0
**Cestoda**							
*Dipylidium caninum*	1 (4) (7–14)	0	1 (3) (0–10)	20 (71) (55–88)	0	0	20 (24) (14–33)
*Spirometra erinacei*	12 (44) (27–62)	6 (50) (25–75)	18 (46) (31–61)	0	0	3 (6) (0–14)	3 (4) (0–8)

^†^ Necropsy samples; * Faecal samples; ^§^ 95% confidence intervals; NI = not investigated.

**Table 2:** Home range areas (MCP100) of 11 tracked dingoes and seven free-roaming domestic dogs.

Dingo/Dog	Sex	Mass (kg) [BCS]	Duration of Tracking (days)	Home Range (km^2^)	Location
**Dingo**					
TD01	Male	27 [5]	79	107.3 *	Walsh’s Pyramid
TD02	Male	21 [5]	195	76.8 *	Mount Peter
TD03	Male	21.5 [4]	120	34.5 *	Glen Boughton
TD04	Female	17 [3]	150	57.1 *	Walsh’s Pyramid
TD06	Female	13 [3]	122	6.9 *	Old Smithfield
TD07	Female	9 [3]	101	8.1 *	Old Smithfield
TD08	Female	13 [3]	171	79.0 *	Walsh’s Pyramid
TD09	Female	15 [4]	202	85.9 *	Glen Boughton
TD10	Female	14.5 [4]	96	26.1 *	Walsh’s Pyramid
TD11	Female	14 [3]	17	5.1	Yarrabah
TD12	Male	14 [4]	58	6.2	Yarrabah
Mean				44.8 (±11.38)	
**Dog**					
CD01	Female (desexed)	14 [5]	67	2.4	Yarrabah
CD02	Male	30 [5]	28	2.7	Yarrabah
CD03	Male	33 [5]	80	2.6	Yarrabah
CD04	Male	32 [5]	15	0.6	Yarrabah
CD05	Male	7 [5]	26	2.2	Yarrabah
CD06	Male	38 [5]	65	0.4	Yarrabah
CD07	Female	30 [5]	27	5.1	Yarrabah
Mean				2.3 (±0.59)	

* Data from concurrent study [26].

## List of Abbreviations

GPS	Global Positioning System
WTWHA	Wet Tropics World Heritage Area
BCS	Body condition score
